# Partial Depletion of Regulatory T Cells Does Not Influence the Inflammation Caused by High Dose Hemi-Body Irradiation

**DOI:** 10.1371/journal.pone.0056607

**Published:** 2013-02-11

**Authors:** Shihong Ma, James A. Richardson, Andrew Bitmansour, Timothy D. Solberg, Rajesh Pidikiti, Kwang Song, Strahinja Stojadinovic, Ellen S. Vitetta, Jeffrey J. Meyer

**Affiliations:** 1 Department of Radiation Oncology, The University of Texas Southwestern Medical Center, Dallas, Texas, United States of America; 2 Cancer Immunobiology Cente, The University of Texas Southwestern Medical Center, Dallas, Texas, United States of America; 3 Department of Pathology, The University of Texas Southwestern Medical Center, Dallas, Texas, United States of America; 4 Department of Microbiology, The University of Texas Southwestern Medical Center, Dallas, Texas, United States of America; 5 Department of Immunology, The University of Texas Southwestern Medical Center, Dallas, Texas, United States of America; University of Nebraska Medical Center, United States of America

## Abstract

There is clinical interest in the modulation of regulatory T cells for cancer therapy. The safety of these therapies in combination with conventional anti-cancer therapies, including radiation therapy, can be studied in animal models. The effects of partial depletion of regulatory T (Treg) cells with an anti-CD25 antibody in conjunction with ionizing radiation on inflammation and tissue injury were analyzed in C57BL/6 mice. An anti-CD25 antibody (PC61) was administered 3 days prior to 13 Gy lower-half hemi-body irradiation (HBI). The blood, spleen, mesenteric lymph nodes (mLNs) and inguinal lymph nodes (iLNs) were harvested at various times thereafter. Alterations in the proportion of leukocyte subsets including CD4^+^ T cells, CD8^+^ T cells, Treg cells, B cells, NK cells, NK1.1^+^ T cells, macrophages and granulocytes were analyzed by FACS. The lungs, liver, pancreas, stomach, jejunum, duodenum, ileum, colon and kidney were harvested and studied by H&E staining. Expression of inflammatory mediators in plasma and tissue were investigated by ELISA. HBI significantly decreased the leukocyte pool though the various leukocyte subsets had different sensitivities to HBI. The administration of PC61 significantly decreased the proportion of Treg cells in spleen, iLN, mLN and blood (reduction of approximately 60%). Irradiation significantly increased the proportion of Treg cells in the spleen, iLN and mLN. HBI induced a systemic inflammatory reaction as demonstrated by increased plasma levels of IL-6, KC/CXCL1 and circulating granulocytes in the blood. Neutrophils also infiltrated the small bowel. The same general patterns were observed whether or not Treg cells were partially depleted with PC61 prior to HBI. These data demonstrate that partial depletion of Treg cells in these mice does not influence HBI-induced inflammatory response and tissue injury, and that combining anti-CD25 therapy with radiation may be safe and well tolerated in a clinical setting.

## Introduction

Radiation therapy (RT) is a first-line treatment option for patients with many different types of solid tumors. Although RT is often very effective at controlling tumors, it can also injure normal tissues. This collateral damage is a particularly critical consideration whenever radiation is combined with other cytotoxic or biological therapies such as chemotherapy and immunotherapy [1].

There is increasing evidence that local tumor irradiation can enhance host anti-tumor immunity [2]. For example, in preclinical studies in mice, high-dose irradiation of tumors resulted in eradication of the primary tumor as well as distant metastases by promoting the maturation of tumor-specific CD8^+^ cytolytic T cells, and increasing their ability to traffic to the tumor site [3], [4]. Irradiation of tumors can enhance the ability of dendritic cells injected intratumorally to capture tumor antigens, migrate to the draining lymph node, and present processed antigens to tumor-specific T cells [5], [6]. Thus, although often viewed as an immunosuppressive treatment modality, by promoting antigen presentation in an inflammatory setting radiation can in fact synergize with antigen-presenting cells to stimulate anti-tumor immunity.

Regulatory T (Treg) cells play a central role in the maintenance of self tolerance and immune homeostasis [7]. In some settings, Treg cells can also suppress anti-tumor responses. The proportion of Treg cells increases in several cancers such as ovarian cancer, non-small cell lung cancer, pancreatic cancer and breast cancer and can inhibit anti-tumor immune responses [8], [9]. Strategies to modulate or deplete Treg cells can enhance anti-tumor immunity, but as expected the depletion of Treg cells can also induce autoimmunity [10], [11]. CD25 is often expressed on Treg cells, and anti-CD25 antibodies are being evaluated in clinical studies in an effort to study their immunomodulating, anti-tumor properties [12].

There is also increasing evidence that Treg cells play an important role in repair of tissue injury from different pro-inflammatory stimuli, including chemotherapy-related injury as well as general trauma, thus broadening their repertoire of activities to include general maintenance of tissue homeostasis [13], [14]. As a result, the depletion and/or inactivation of Treg cells in combination with tumor-damaging therapies such as chemotherapy and radiation may lead to synergistic interactions and tumor rejection, but at the possible price of uncontrolled inflammation and enhanced normal tissue injury by inflammatory and autoimmune mechanisms. The interaction between radiation and Treg cells is a new area of study with much of the focus on anti-tumor effects [15]–[17]. Kachikwu *et al* [16] have shown promising anti-tumor efficacy following combination of an anti-CD25 antibody with radiation, but detailed analysis of normal tissue injury resulting from this combination is not yet available.

The possible exacerbation of normal tissue injury when combining Treg cell depletion with radiation is an important preclinical issue that can be explored prior to undertaking clinical studies. To address this issue, we have compared inflammatory markers and tissue injury in C57BL/6 mice after high-dose hemi-body irradiation (HBI) and/or HBI in combination with monoclonal antibody (MAb)-based partial depletion of Treg cells with anti-CD25. Our results indicate that partial depletion of Treg cells with this antibody does not significantly influence the inflammation and injury induced by high-dose irradiation alone.

## Materials and Methods

### Ethics Statement

Following the guidelines from the National Institute of Health, the use of mice was approved by the Institutional Animal Care Use Center of the University of Texas Southwestern Medical Center (UTSW, Dallas, TX). All animal protocols were approved by the Institutional Review Board. The approval animal protocol number (APN) was 2010-0142.

### Mice

Female C57BL/6 mice (6–8 wk old) were purchased from Taconic (Hudson, NY). Following the guidelines from the National Institute of Health, these animals were housed and cared for in the pathogen-free facility of the Institutional Animal Care Use Center of the University of Texas Southwestern Medical Center (UTSW, Dallas, TX). All animal protocols were approved by the Institutional Review Board.

### Antibodies

The hybridoma cells producing anti-mouse CD25 (clone PC61, rat IgG1) MAb were obtained from the American Type Culture Collection (ATCC, Manassas, VA). Culture supernatants were collected and PC61 was purified by affinity chromatography on Sepharose-Protein G. The rat serum IgG control was obtained from Sigma-Aldrich (St. Louis, MO) and has been used as a control in other studies with these antibodies [18], [19].

For flow cytometry staining, anti-CD4-FITC (clone GK1.5), biotin-conjugated anti-CD25 (clone 7D4), anti-FoxP3-PE (clone FJK-16s), anti-CD45R (B220)-PE-Cy5 (clone RA3-6B2), anti-CD11b-PE-Cy5 (clone M1/70, Mac-1α subunit), anti-CD3-APC (clone 145-2C11), anti-CD8-PE-Cy5 (clone 53-6.7), anti-CD19-APC (clone eBio1D3), anti-NK1.1-PE (clone PK136), anti-F4/80 Antigen-PE (clone BM8) and anti-Ly-6G (Gr-1)-FITC (clone RB6-8C5) were obtained from eBioscience (San Diego, CA). Biotin-conjugated anti-CD25 was used together with streptavidin-APC (Jackson ImmunoResearch Laboratories, West Grove, PA).

### Treg cell depletion and irradiation

**Figure 1:**
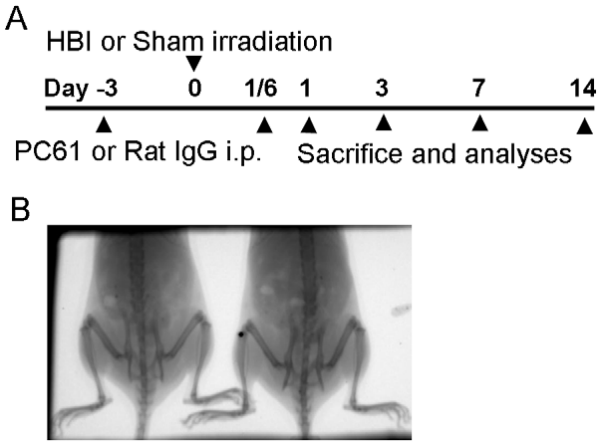
Treatment Schedules. (A) Schedule for Treg cell depletion and irradiation. Three days prior to irradiation, mice were administered 0.75 mg PC61 to deplete Treg cells. On day 0, mice were irradiated to 13 Gy to the lower-half hemi-body (hemi-body irradiation: HBI). Mice were sacrificed at 4 hrs, 1, 3, 7 and 14 days after radiation exposure. (B) Irradiation target: the abdomen and pelvis of mice.

C57BL/6 mice were randomized into four groups: (i) Control: Sham HBI+0.75 mg Rat IgG, (ii) Treg cell depletion: Sham HBI+0.75 mg PC61, (iii) Radiation: 13 Gy HBI+0.75 mg Rat IgG, (iv) Radiation plus Treg cell depletion: 13 Gy HBI+0.75 mg PC61. Treg cells were depleted with a single i.p. injection 0.75 mg of PC61 MAb on day −3 [20]. Control groups received an i.p. injection of 0.75 mg normal Rat IgG at identical time points (Figure 1A).

A dose of 13 Gy was delivered in a single fraction using a dedicated x-ray irradiator (X-RAD 320, Precision X-Ray, Inc., North Branford, CT). The device was operated at 250 kV and 1 mA, producing a dose rate of 1.27 Gy/min. Dosimetric calibration of the device was performed according to national protocols and has been described previously [21]. The small animal irradiator is equipped with a portable gas anesthesia device. During the irradiation procedure, mice were maintained under anesthesia by inhalation of 1% isoflurane gas in air at 1 dm^3^/min through a nose cone attached to the support bed. Abdominal irradiation was performed through a single dorsal-ventral field, and two mice were irradiated at a time using a custom 25.4 mm thick brass 7 cm×3 cm collimator as shown in Figure 1B. Image guidance was used for localization to ensure accurate targeting of the abdomen. The imaging device was developed by the Division of Medical Physics and Engineering at our institution; its operation has been described previously [22]. Sham-irradiated mice were treated in an identical manner but were not exposed to the radiation source.

Mice were continuously monitored for activity, and weighed until they were euthanized at 4 hours (hrs) and 1, 3, 7 and 14 days after treatment. Peripheral blood, inguinal lymph nodes (iLNs), mesenteric lymph nodes (mLNs) and spleens were collected for analysis. CD25 was detected using an anti-CD25 (clone 7D4) that recognizes an epitope distinct from that recognized by the PC61 MAb [23]. Plasma was harvested to measure cytokine levels. To observe abnormalities in tissues, lung, liver, pancreas, stomach, jejunum, duodenum, ileum, colon and kidney were harvested, fixed in 4% paraformaldehyde and embedded in paraffin. Sections were stained with hematoxylin and eosin.

### Staining and flow cytometry

Cells were stained with relevant MAbs for 30 minutes on ice and were washed. FoxP3 was detected as described previously [24] using a staining buffer set from eBioscience (San Diego, CA) for fixation and permeabilization of cells. Fluorescence intensities of the stained cells were analyzed using a FACSCalibur flow cytometer (BD Biosciences, San Jose, CA). Data were analyzed using FlowJo software (Tree Star, Inc., Ashland, OR).

For phenotyping, spleens, iLNs, mLNs and blood cells were harvested and examined for the proportions of CD4^+^ T cells (CD3^+^CD4^+^), CD8^+^ T cells (CD3^+^CD8^+^), B cells (CD19^+^), NK cells (CD3^-^NK1.1^+^), NK1.1^+^ T cells (CD3^+^NK1.1^+^), macrophages (CD11b^+^F4/80^+^) and granulocytes (CD11b^+^GR1^+^) using the appropriate MAbs. The total numbers of cells and lymph nodes were also determined. The extent of Treg cell (CD4^+^CD25^+^FoxP3^+^) depletion and the dynamics of Treg cell repopulation were evaluated.

### Measurement of inflammatory mediators

Mice were sacrificed at the indicated time points and blood was obtained by cardiac puncture and collected in tubes containing EDTA. Plasma were collected after centrifugation, aliquoted and stored at −20 °C for cytokine and chemokine KC/CXCL1 analysis.

The levels of IL-1β, IL-6, IL-10, KC/CXCL1, TNF-α, IFN-γ and TGF-β in plasma were determined by ELISA kits according to the manufacturer's protocol (R&D Systems, Minneapolis, MN). Measurements of cytokines in jejunum, duodenum, ileum and lung were carried out using the same assay kit. Tissues were homogenized in phosphate buffer containing a protease inhibitor cocktail (diluted 1:100, Sigma-Aldrich, St. Louis, MO) using a TissueLyser II (Qiagen, Valencia, CA). Samples were then centrifuged and the supernatants were evaluated for protein and cytokine content. Total protein concentrations were determined using Coomassie protein assay (Sigma-Aldrich, St. Louis, MO) with BSA as a standard.

### Histological examination

Lungs, liver, pancreas, stomach, jejunum, duodenum, ileum, colon and kidney were fixed in 4% paraformaldehyde, thin sectioned and studied with H&E staining by members of our Pathology Core Laboratory. Histological examination was carried out using a Leica DM2000 microscope at a magnification of 100× or 200×. Photos were captured using a microscope equipped with Micro FIRE™ camera (Optronics, Goleta, CA). Images were analyzed by the PictureFrame® image-processing software (Optronics, Goleta, CA).

### Statistical analysis

The interactions between time and treatment groups were analyzed by two-way analysis of variance (ANOVA). Tukey's HSD (honesty significant difference) was used to compare groups with multiple comparisons (post hoc tests). These analyses were performed using SPSS 18.0 software. Differences were considered to be statistically significant when p<0.05. All experiments were performed at least 3 times with representative results illustrated.

## Results

### Systemic effects of hemi-body irradiation with and without depletion of Treg cells

**Figure 2:**
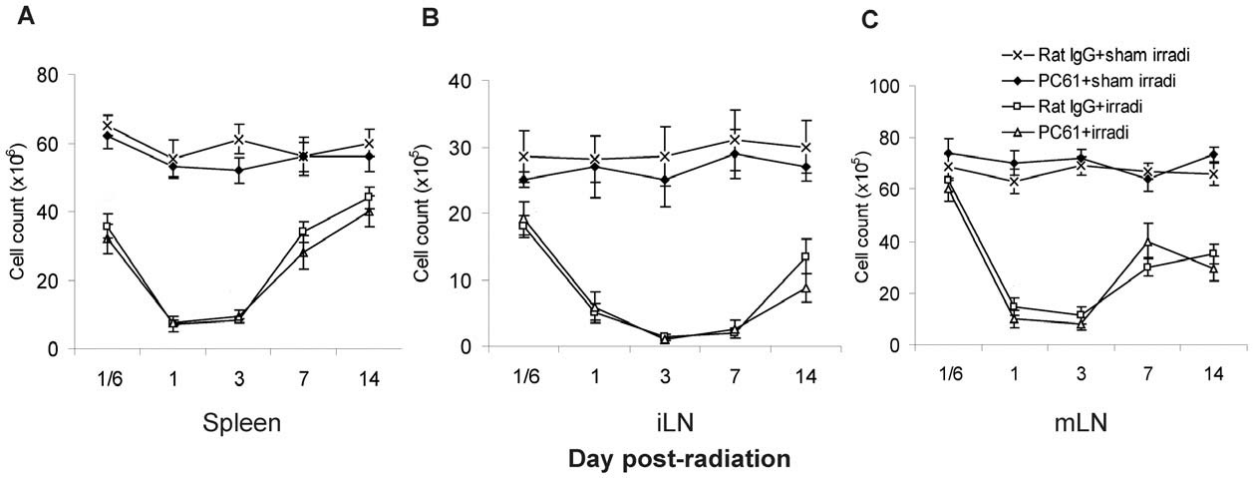
Cell numbers in the spleens and lymph nodes. Mice were injected with PC61 or rat IgG MAb 3 days prior to receiving 13 Gy of HBI or sham irradiation. Cells in the (A) spleen, (B) iLN and (C) mLN were counted on the days indicated. Data are shown as Mean±SD (n = 4). Two way ANOVA showed significant interactions over time between treatments in spleen, iLN and mLN cell count (all p<0.01). No differences were detected between PC61+irradiation compared to Rat IgG+irradiation treatment group profiles over time in the spleen, iLN and mLN (p≥0.128). No significant differences were found between the PC61+sham irradiation compared to Rat IgG+sham irradiation treatment group profiles over time (all p≥0.072). Differences between all other pair-wise comparisons of treatment group profiles over time were significant (p<0.05). All post hoc pairwise comparisons were performed with Tukey's multiple comparisons. This is one representative experiment of three performed.

To determine the effects of radiation with and without the depletion of Treg cells on systemic leukocyte counts, alterations in the total number of cells in the spleen, iLN and mLN, as well as alterations in the proportions of various leukocyte subsets were determined at 4 hrs and 1, 3, 7 and 14 days following irradiation. Radiation significantly decreased the number of total spleen, iLN and mLN cells in the mice treated with both HBI and rat IgG and HBI+PC61. Cell numbers decreased as early as 4 hrs, reached a nadir of about 10–15% of the normal control levels 3 days following irradiation, and gradually increased thereafter (Figure 2). Depletion of Treg cells with PC61 did not substantially influence HBI-induced decreases in the number of leukocytes in the spleen and LNs

**Figure 3:**
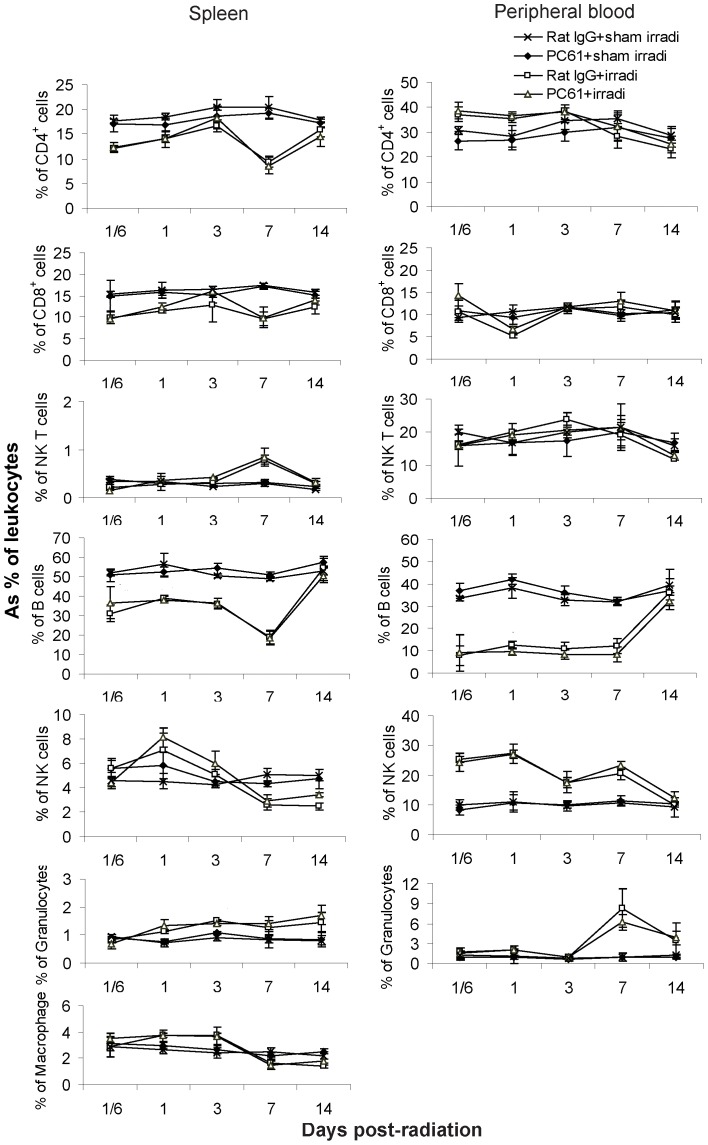
Leukocyte subsets in the spleen and blood. In spleen, irradiation reduced proportions of B cells, CD4^+^ T cells and CD8^+^ T cells; increased proportions of NK1.1^+^ T cells and granulocytes. In blood, B cells were most radiosensitive; NK cells were most resistant; granulocytes increased on days 7 and 14. Data are expressed as Mean±SD (n = 4). Two way ANOVA showed significant interactions over time between treatments in all cell subsets (p<0.01) except NK1.1^+^ T cells in blood (p = 0.288). No significant differences were detected between PC61+irradiation compared to Rat IgG+irradiation treatment group profiles over time for any of the subsets in the spleen (p≥0.322) and blood (p≥0.095). No significant differences were found between the PC61+sham irradiation compared to Rat IgG+sham irradiation treatment group profiles over time (all p>0.05). Differences between all other pair-wise comparisons of treatment group profiles over time were significant (p<0.05). All post hoc pairwise comparisons were performed with Tukey's multiple comparisons. This is one representative experiment of three performed.

The proportion of splenic B cells, CD4^+^ T cells and CD8^+^ T cells decreased while NKT cells, granulocytes and macrophages increased after HBI. Also, B cells decreased even more dramatically in the blood while the proportion of NK cells and granulocytes increased following irradiation (Figure 3). There were no differences in the depletion of specific leukocyte subsets in mice undergoing HBI alone *vs* HBI+depletion of Treg cells. In general, B cells were the most radiosensitive, CD4^+^ T cells and CD8^+^ T cells were moderately sensitive, while NKT cells, macrophages and granulocytes were relatively radioresistant. NK cells were the most radioresistant.

Irradiated mice also experienced ∼5% weight loss on days 1 and 3 (Supplementary Table S1). None of the irradiated mice died during the 3 month observation period. Depletion of Treg cells with PC61 did not influence HBI-induced weight loss.

### The response of Treg cells to hemi-body irradiation with and without anti-CD25 antibody therapy

**Figure 4:**
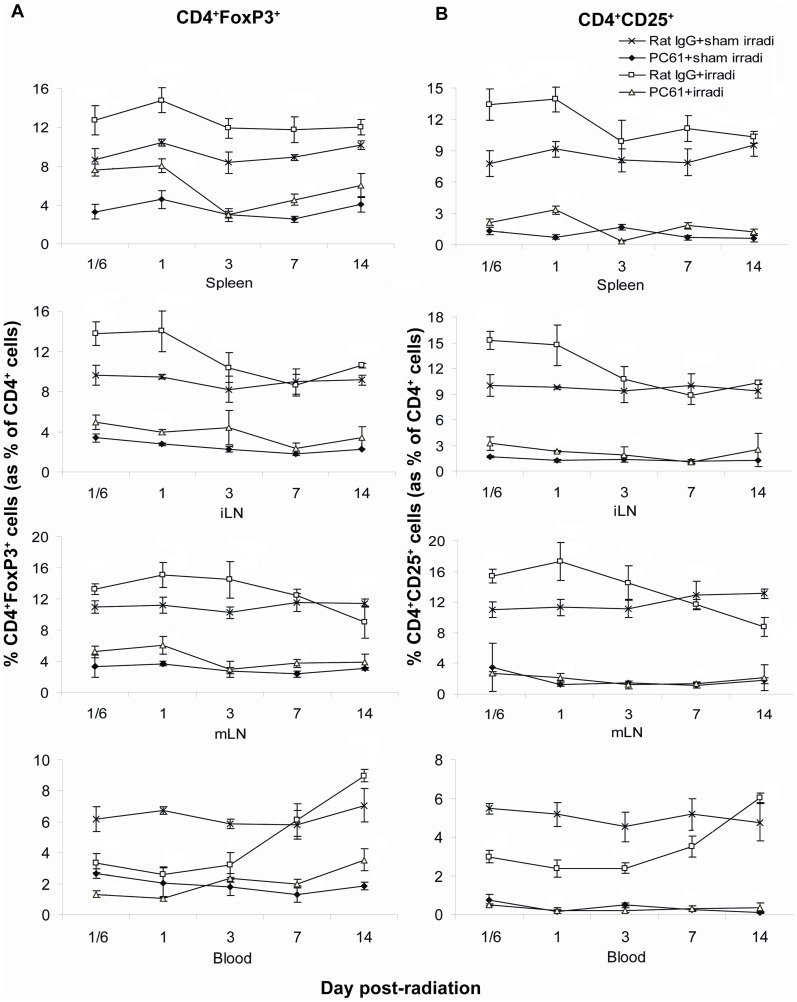
The proportion of CD4^+^FoxP3^+^ cells in the spleens, LNs and peripheral blood. (A) The proportion of CD4^+^FoxP3^+^ Treg cells and (B) the proportion of CD4^+^CD25^+^ cells. PC61 administration reduced the proportion of CD4^+^FoxP3^+^ Treg cells and CD4^+^CD25^+^ cells in spleen, iLN, mLN and blood, irradiation increased their proportion in spleen, iLN, and mLN but not in blood. Data are shown as Mean±SD (n = 4). Two way ANOVA showed significant interactions over time between treatments in the proportion of CD4^+^FoxP3^+^ Treg cells and in the proportion of CD4^+^CD25^+^ cells in all compartments (all p<0.01). Differences between all pairs (PC61+sham irradiation *vs* Rat IgG+sham irradiation, PC61+irradiation *vs* PC61+sham irradiation, Rat IgG+sham irradiation compare to Rat IgG+irradiation, Rat IgG+sham irradiation compare to PC61+irradiation, PC61+sham irradiation compared to Rat IgG+irradiation and PC61+sham irradiation compared to PC61+irradiation treatment groups) of treatment group profiles over time were significant (p<0.05) except the proportion of Treg cells between PC61+sham irradiation *vs* PC61+irradiation in blood (p = 0.948), and the proportion of CD4^+^CD25^+^ cells between PC61+sham irradiation *vs* PC61+irradiation in all compartments (p≥0.105). All post hoc pairwise comparisons were performed with Tukey's multiple comparisons. This is one representative experiment of three performed.

To characterize the effect of radiation on Treg cells, the proportion of CD4^+^FoxP3^+^ cells within the total CD4^+^ cell population was evaluated following HBI+/−treatment with PC61. As expected, the administration of PC61 significantly decreased the proportion of Treg cells in spleen, iLN, mLN and blood, although this depletion was incomplete (Figure 4). Depletion of Treg cells with PC61 alone reached its nadir on day 7 with a relative reduction of ∼60%. Compared to sham irradiation, irradiation alone caused a significant increase in the proportion of Treg cells in the spleen, iLN and mLN, and a decrease in their proportion in the blood at 4 hrs, and 1 and 3 days. PC61+HBI treatment resulted in an increase in the proportion of Treg cells in the spleen, as compared with PC61+sham irradiation treatment. However, the proportion of Treg cells in mice treated with PC61+HBI was significantly lower than that of the mice receiving Rat IgG+HBI. Overall, the changes in the proportion of the CD4^+^CD25^+^ subset within the CD4^+^ cell population were similar to those of CD4^+^FoxP3^+^ cells (Figure 4).

**Figure 5:**
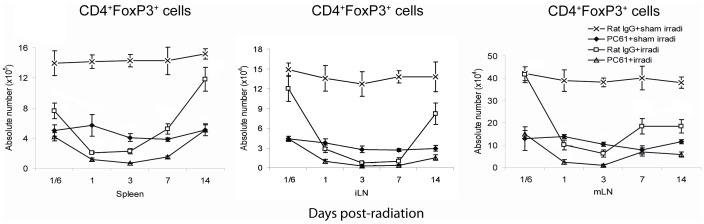
The absolute number of CD4^+^FoxP3^+^ Treg cells in the spleens and LNs. Both PC61 administration and irradiation reduced the number of CD4^+^FoxP3^+^ Treg cells in spleen, iLN and mLN. Irradiation resulted in a more rapid restoration of Treg cells after day 7. Data are shown as Mean±SD (n = 4). Two way ANOVA showed significant interactions over time between treatments in the Treg absolute numbers in spleen, iLN and mLN (all p<0.01). Differences between all pairs (PC61+sham irradiation *vs* Rat IgG+sham irradiation, PC61+irradiation *vs* PC61+sham irradiation, Rat IgG+sham irradiation compare to Rat IgG+irradiation, Rat IgG+sham irradiation compare to PC61+irradiation, PC61+sham irradiation compared to Rat IgG+irradiation and PC61+sham irradiation compared to PC61+irradiation treatment groups) of treatment group profiles over time were significant (p<0.05). All post hoc pairwise comparisons were performed with Tukey's multiple comparisons. This is one representative experiment of three performed.

In the radiation-treated mice, the absolute numbers of Treg cells decreased over the first week and repopulated quickly thereafter in spleen and iLN. PC61+HBI treatment resulted in the most significant decrease in the number of Treg cells, with recovery incomplete by day 14 (Figure 5).

### Markers of inflammation: plasma

**Figure 6:**
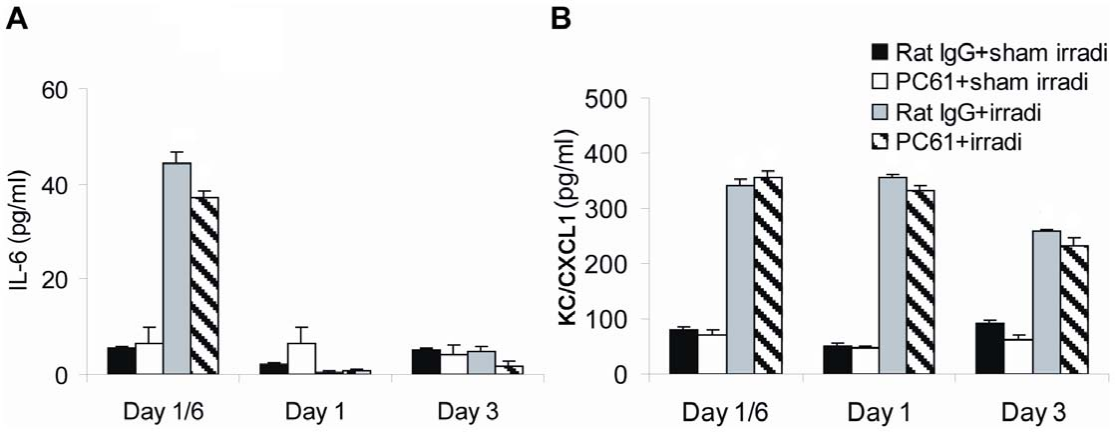
Inflammatory mediators in plasma. Plasma levels of (A) IL-6 and (B) KC/CXCL1 were determined at different time points after treatment. Data are shown as Mean±SD (n = 4). Two way ANOVA showed significant interactions over time between treatments (all p<0.01). No differences were detected between PC61+irradiation compared to Rat IgG+irradiation treatment group profiles over time in the levels of IL-6 (p≥0.099) and KC/CXCL1 (p≥0.475). No significant differences were found between the PC61+sham irradiation compared to Rat IgG+sham irradiation treatment group profiles over time (all p>0.05). Differences between all other pair-wise comparisons of treatment group profiles over time were significant (p<0.05). All post hoc pairwise comparisons were performed with Tukey's multiple comparisons. This is one representative experiment of three performed.

To evaluate the systemic inflammatory response to radiation+/−depletion of Treg cells, inflammatory mediators in the plasma and the proportion of circulating granulocytes were determined at 4 hrs and days 1, 3 7 and 14 following irradiation. Four hours after radiation exposure, plasma levels of IL-6 and KC/CXCL1 significantly increased. At one and three days following irradiation, IL-6 levels returned to control levels while KC/CXCL1 remained significantly elevated (Figure 6). At 7 and 14 days following irradiation, levels of KC/CXCL1 returned to control levels (data not shown). Identical patterns were observed in irradiated mice in which Treg cells were depleted with PC61. Levels of IL-1β, IL-10, TNF-α, IFN-γ and TGF-β were undetectable in the plasma in all four groups of mice throughout the time course studied (data not shown). The percentages of circulating granulocytes were increased at 7 and 14 days following irradiation as shown in Figure 3; this was not influence by treatment with PC61.

### Markers of inflammation: tissues

**Figure 7:**
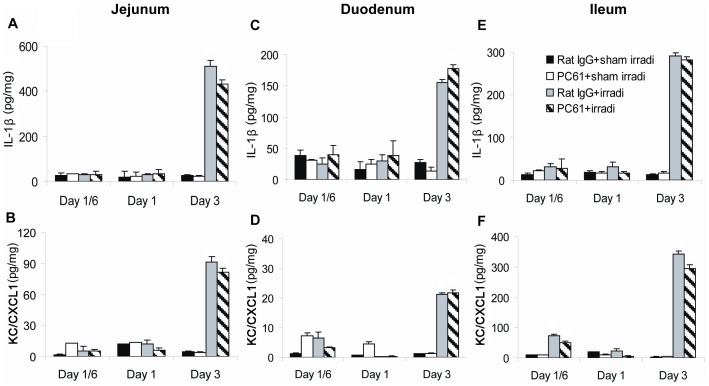
Production of inflammatory mediators in the jejunum, duodenum and ileum. Jejunum, duodenum and ileum were collected and levels of IL-1β and KC/CXCL1 were measured by ELISA of tissue lysates. (A) IL-1β and (B) KC/CXCL1 in jejunum, (C) IL-1β and (D) KC/CXCL1 in duodenum, (E) IL-1β and (F) KC/CXCL1 in ileum. Data are shown as Mean±SD (n = 4). Two way ANOVA showed significant interactions over time between treatments (all p<0.01). No differences were detected between PC61+irradiation compared to Rat IgG+irradiation treatment group profiles over time in the levels of IL-1 β (p≥0.119) and KC/CXCL1 (p≥0.084). No significant differences were found between the PC61+sham irradiation compared to Rat IgG+sham irradiation treatment group profiles over time (all p>0.05). Differences between all other pair-wise comparisons of treatment group profiles over time were significant (p<0.05). All post hoc pairwise comparisons were performed with Tukey's multiple comparisons. This is one representative experiment of three performed.

Levels of IL-1β, IL-6, IL-10, KC/CXCL1 and TNF-α were measured in lysates from the duodenum, jejunum, ileum, colon and lung. Three days following HBI, the levels of IL-1β and KC/CXCL1 in the duodenum, jejunum and ileum of irradiated mice increased (Figure 7). The levels of IL-6 and KC/CXCL1 returned to control levels at 7 and 14 days following HBI (data not shown). However, IL-6, IL-10 and TNF-α were not detected in the intestines (data now shown). Moreover, none of these inflammatory mediators were detected in the colon (data not shown).

**Figure 8:**
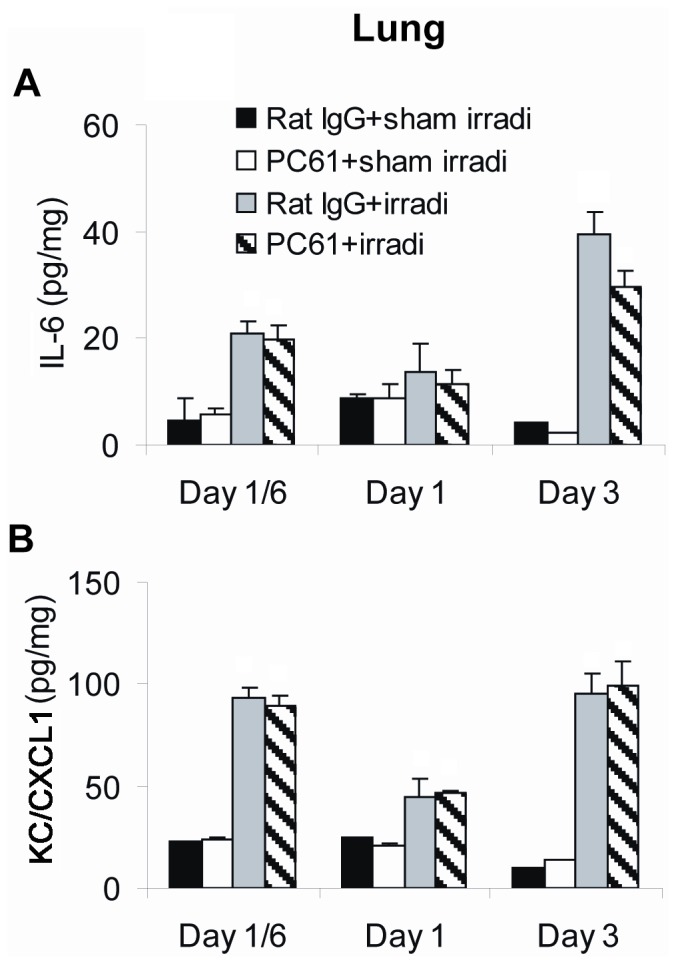
Production of inflammatory mediators in the lungs. Levels of (A) IL-6 and (B) KC/CXCL1 were measured in lysates of lung by ELISA. Data are shown as Mean±SD (n = 4). Two way ANOVA showed significant interactions over time between treatments (all p<0.01). No differences were detected between PC61+irradiation compared to Rat IgG+irradiation treatment group profiles over time in the levels of IL-6 and KC/CXCL1 (p≥0.089). No significant differences were found between the PC61+sham irradiation compared to Rat IgG+sham irradiation treatment group profiles over time (all p>0.05). Differences between all other pair-wise comparisons of treatment group profiles over time were significant (p<0.05). All post hoc pairwise comparisons were performed with Tukey's multiple comparisons. This is one representative experiment of three performed.

In addition to increased pro-inflammatory cytokines in irradiated small intestine, levels of IL-6 and KC/CXCL1 were significantly increased in the lungs 3 days following radiation exposure. The level of KC/CXCL1 was elevated as early as 4 hrs after exposure (Figure 8). This may have been caused by an out-of-field effect of radiation or by radiation scatter. Levels of IL-1β, IL-10 and TNF-α were undetectable in the lung (data not shown). Results were identical in irradiated mice in which Treg cells were depleted. Thus, in these mice, radiation did indeed induce an inflammatory cytokine response and depletion of Treg cells did not influence the intensity or course of this response.

### Tissue injury

**Figure 9:**
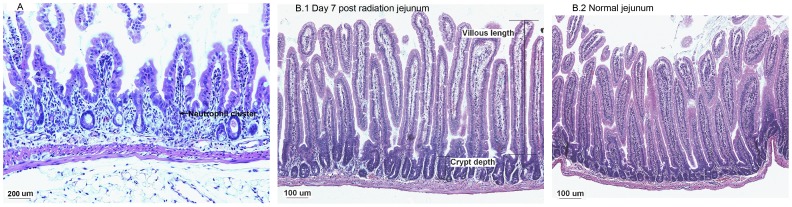
GI injury. Tissues were fixed, thin sectioned and studied with H&E staining. (A) Neutrophil infiltration in jejunum at 3 day after radiation. 200× magnification. The arrow on the image shows a neutrophil cluster. 200× magnification. (B) Crypt hyperplasia at 7 days after radiation. 100× magnification.

It is well known that radiation causes tissue damage in the gastrointestinal (GI) tract. To determine whether depletion of Treg cells amplified radiation-induced tissue injury, the lungs, liver, pancreas, stomach, jejunum, duodenum, ileum, colon and kidney were subjected to histological analysis. The most significant tissue damage was observed in the small intestine. In accordance with increased levels of IL-1β and KC/CXCL1 in the small intestine, neutrophil infiltration was observed in the lamina propia 3 days following irradiation (neutrophils identified by morphology). A representative image is shown in Figure 9A. The number of neutrophils in the lamina propria also increased significantly following irradiation (Table 1).

**Table 1:** Number of neutrophils in the lamina propria per high power field (400×).

Group	GI track	Neutrophils (No.)/field		
		Day 1/6	Day 1	Day 3
Rat IgG+sham irradiation	Duodenum	0.7±0.6	0.4±0.7	0.6±0.7
	Jejunum	0.6±0.7	0.3±0.5	1.3±0.9
	Ileum	0.5±0.5	0.5±0.9	1.0±0.9
PC61+ sham irradiation	Duodenum	0.6±0.7	0.4±0.7	0.3±0.5
	Jejunum	0.7±0.8	0.8±0.6	0.6±0.7
	Ileum	0.3±0.5	0.5±0.5	0.3±0.5
Rat IgG+irradiation	Duodenum	0.9±0.5	0.5±0.7	3.6±3.3
	Jejunum	0.7±0.6	0.3±0.5	17±6.3
	Ileum	0.5±0.5	0.4±0.5	21±11
PC61+irradiation	Duodenum	0.6±0.7	0.3±0.5	4.5±3.7
	Jejunum	0.4±0.5	0.4±0.7	15±9.5
	Ileum	0.5±0.9	0.6±0.7	23±7.4

Note: H&E stained tissues were observed under a microscope. The number of neutrophils was calculated in 10 random fields at 400× magnification. Data are expressed as Mean±SD. Two way ANOVA showed significant interactions over time between treatments (all p<0.01). No differences were detected between PC61+irradiation compared to Rat IgG+irradiation treatment groups (p≥0.834). No significant differences were found between groups treated with PC61+sham irradiation compared to Rat IgG+sham irradiation (all p>0.05). Differences between all other pair-wise comparisons of treatment group profile over time were significant (p<0.05). All post hoc pairwise comparisons were performed with Tukey's multiple comparisons.

At 7 and 14 days following irradiation, hyperplasia in the crypts of the small intestine was observed. Villous length, crypt depth and number of crypt branching were used as indicators for crypt hyperplasia. The small intestines from mice receiving radiation treatment showed increased villous length, crypt depth and number of crypt branchings (Figure 9B.1 and B.2 and Table 2). Depletion of Treg cells did not appreciably change the neutrophil influx nor the crypt repair in response to irradiation. No significant differences were observed in other tissues including lung, liver, pancreas, stomach, colon and kidney compared with control mice.

**Table 2:** GI tract hyperplasia at 7 and 14 days after radiation treatment.

Group	GI track	Villous length (µm)		Crypt depth (µm)		Crypt branching (no./field)	
		Day 7	Day 14	Day 7	Day 14	Day 7	Day 14
Rat IgG+sham irradiation	Duodenum	430±20	460±20	80±7	80±7	0.3±0.4	0.4±0.5
	Jejunum	420±20	450±20	70±9	70±4	0.4±0.5	0.1±0.3
	Ileum	310±30	310±20	90±7	80±4	0.3±0.4	0.1±0.3
PC61+sham irradiation	Duodenum	430±30	470±40	70±7	70±4	0.3±0.5	0.3±0.4
	Jejunum	450±10	460±20	80±10	80±4	0.1±0.3	0.2±0.4
	Ileum	300±10	330±20	100±5	80±5	0.2±0.4	0.1±0.3
Rat IgG+irradiation	Duodenum	500±20	470±10	120±10	120±8	1.7±0.7	1.7±0.7
	Jejunum	530±60	390±10	110±7	100±5	1.7±0.9	1.8±0.9
	Ileum	340±20	280±10	100±5	100±7	1.3±0.7	0.8±0.4
PC61+irradiation	Duodenum	560±70	530±40	110±4	120±8	1.5±0.5	1.3±0.7
	Jejunum	560±40	400±10	110±10	110±8	1.0±0.6	1.5±0.5
	Ileum	340±20	310±20	110±7	110±7	0.8±0.7	1.8±1.1

Note: H&E stained tissues were observed under a microscope with 100× magnification. Villous length and crypt depth was measured, no. of crypt branching were calculated in ten random fields. Data are expressed as Mean±SD. For villous length, two way ANOVA showed significant interactions over time between treatments in jejunum and ileum (all p<0.01), no interaction over time between treatments in duodenum (p = 0.058). No differences were detected between PC61+irradiation compared to Rat IgG+irradiation treatment groups (p≥0.059). No significant differences were found between groups treated with PC61+sham irradiation compared to Rat IgG+sham irradiation (all p>0.05). Differences between all other pair-wise comparisons of treatment group profile over time were significant (p<0.05). For crypt depth, two way ANOVA showed no interactions over time between treatments (p≥0.108). No differences were detected between PC61+irradiation compared to Rat IgG+irradiation treatment groups (p≥0.174). No significant differences were found between groups treated with PC61+sham irradiation compared to Rat IgG+sham irradiation (all p>0.05). Differences between all other pair-wise comparisons of treatment group profile over time were significant (p<0.05). For crypt branching, two way ANOVA showed significant interactions over time between treatments (p<0.05), no interactions over time between treatments in duodenum (p = 0.868) and jejunum (p = 0.463). No differences were detected between PC61+irradiation compared to Rat IgG+irradiation treatment groups (p≥0.249). No significant differences were found between groups treated with PC61+sham irradiation compared to Rat IgG+sham irradiation (all p>0.05). Differences between all other pair-wise comparisons of treatment group profile over time were significant (p<0.05). All post hoc pairwise comparisons were performed with Tukey's multiple comparisons.

## Discussion

Stereotactic body radiation therapy, characterized by very high and focal doses of irradiation, has shown dramatic improvements in tumor control relative to more conventional radiation treatments [25], [26]. However, our knowledge of the responses of normal tissues following high dose radiation therapy, especially when combined with immune-modulating methods, is lacking. This is an important area of inquiry because of the growing interest in combining radiation with immunotherapies with the purpose of amplifying host anti-tumor immune responses [27]. In the present study, we investigated the effects of partial Treg cell depletion with anti-CD25 antibody therapy on the inflammatory response and tissue injury of mice subject to 13 Gy of HBI. We studied 4 parameters after mice were treated with high-dose HBI alone or HBI+Treg depletion using a monoclonal anti-CD25 antibody (clone PC61). First, we determined the effects of these treatments on leukocyte subsets in the spleen, LNs and blood. Second, we studied how these treatments influenced the population of Treg cells. Third, the production of pro-inflammatory mediators in the plasma and small intestine was measured following radiation, with and without Treg cell depletion. Finally, a histological analysis of critical organs was carried out to evaluate tissue injury in response to these combinatorial treatments.

The major observations to emerge from this study are as follows: 1. Leukocyte subsets have different sensitivities to RT. Similar findings have been reported by other authors [28]–[30]; however, in our study, we demonstrate that depletion of Treg cells does not substantively affect this outcome. 2. The proportion of Treg cells increases in response to HBI, representing either increased resistance to RT relative to other T cell subsets or as a host response to the injury induced by RT. 3. HBI induces transient systemic and local inflammation, and partial depletion of Treg cells by PC61 did not alter the systemic and local inflammatory responses to HBI.

Prior studies have shown that Treg cells play an important role in host response to tissue injury. Treg cells appear to play a role in responding to diverse injuries, in addition to their well-known role in suppressing adaptive immune responses [13], [14]. Therapies that deplete Treg cells (even partially), could also amplify radiation-induced inflammation and injury. The aim of our study was to determine whether treatment with PC61 plus high-dose hemi-body irradiation enhanced injury as compared to treatment with radiation alone. Our results indicate that the partial depletion of Treg cells using the PC61 MAb did *not* increase radiation-induced injury and inflammation.

There are a number of possibilities to explain these results. First, Treg cells may not be an important component of the host homeostatic response to radiation-induced tissue damage. Radiation alone depletes Treg cell numbers. Other cellular mechanisms may be more important. Studies with adoptive transfer of Treg cells might further delineate their role in repair of radiation-induced injury. Secondly, if Treg cells do play this role, the radiation dose used in our studies, 13 Gy, may not have induced sufficient inflammatory damage to cause a major Treg cell response. We chose this radiation dose since higher doses are associated with mortality from the gastrointestinal syndrome. Future studies can evaluate more focal irradiation of portions of select organs, such as the liver, to higher doses which will not, by itself, lead to death (and where radiation-induced decreases in the numbers of Treg cells could be less significant than in our current model). Treg cells may indeed play a role in the homeostatic response of tissues to even higher doses of irradiation. Third, although PC61 does indeed decrease of numbers of CD4^+^FoxP3+ Treg cells, the depletion is not complete, as has been shown in other studies. Complete depletion of Treg cells may yield different results. Fourth, PC61 may also deplete effector, non-regulatory T cells [20]. Finally, mouse strain and/or environmental differences in tissue response to radiation may account for our results. Performing a similar study in other mouse strains may yield different results.

To date, there is no single specific antibody or small molecule inhibitory drug agent that selectively targets and depletes Treg cells. Nonetheless, PC61 is widely used in the study of Treg cell biology, and prior studies have shown that even modest decreases in the numbers/proportions of Treg cells are sufficient to produce both autoimmune events as well as anti-tumor immune responses. Radiation in conjunction with PC61 has been reported to induce anti-tumor immunity in model tumor systems. Moreover, there is already clinical evidence that CD25-targeting antibodies in humans, such as denileukin diftitox, may stimulate anti-tumor immune responses, and this type of immunotherapy is under investigation in clinical trials [12]. There can be little doubt that perturbations in the numbers of Treg cells can have significant consequences in several settings.

There is great interest in modulating regulators of the immune system. Although anti-tumor responses can be observed by manipulating Treg cells, the risk of significant autoimmune reactions could tip the therapeutic benefit. Combining various immune-modulating drugs with tumor-ablating treatments such as thermal ablation or radiation holds great promise, but again with potentially significant costs to the patients that may exceed the risks associated with the drug itself, particularly when there is a risk for ‘synergistic toxicity’ by combining the two treatments. We have established a simple normal tissue injury model where variables such as the specific immune-modulating drug, radiation dose, and the volume of irradiated tissue can be easily adjusted. This model system should serve as a useful preclinical model to study the safety of combinations of radiation with immunotherapies.

In summary, in our model system-, partial depletion of Treg cells with the anti-CD25 antibody, PC61 did not amplify HBI-induced systemic/local inflammatory responses or tissue injury. This suggests that modulating levels of Treg cells in conjunction with radiation therapy might synergize to eradicate tumors without causing enhanced normal tissue injury.

## Supporting Information

Table S1 **Percentage of body weight change after irradiation and/or Treg cell depletion.**
(DOC)

## References

[1] Paganetti H, Yock T, Tarbell NJ, Trofimov A (2011) Optimization of radiotherapy treatment delivery technology to minimize radiation injury. In: Shrieve DC, Loeffler JS, editors. Human radiation injury. Philadelphia: Lippincott Williams & Wilkins. pp. 114–125.

[2] Nesslinger NJ, Sahota RA, Stone B, Johnson K, Chima N, et al (2007) Standard treatments induce antigen-specific immune responses in prostate cancer. Clin Cancer Res 13: 1493–1502. 17332294 10.1158/1078-0432.CCR-06-1772

[3] Lee Y, Auh SL, Wang Y, Burnette B, Meng Y, et al (2009) Therapeutic effects of ablative radiation on local tumor require CD8+ T cells: changing strategies for cancer treatment. Blood 114: 589–595. 19349616 10.1182/blood-2009-02-206870 PMC2713472

[4] Lugade AA, Moran JP, Gerber SA, Rose RC, Frelinger JG, et al (2005) Local radiation therapy of B16 melanoma tumors increases the generation of tumor antigen-specific effector cells that traffic to the tumor. J Immunol 174: 7516–7523. 15944250 10.4049/jimmunol.174.12.7516

[5] Teitz-Tennenbaum S, Li Q, Okuyama R, Davis MA, Sun R, et al (2008) Mechanisms involved in radiation enhancement of intratumoral dendritic cell therapy. J Immunother 31: 345–358. 18391761 10.1097/CJI.0b013e318163628c PMC3103774

[6] Moyer JS, Li J, Wei S, Teitz-Tennenbaum S, Chang AE (2008) Intratumoral dendritic cells and chemoradiation for the treatment of murine squamous cell carcinoma. J Immunother 31: 885–895. 18832999 10.1097/CJI.0b013e3181880f1e PMC4078665

[7] Wing K, Fehervari Z, Sakaguchi S (2006) Emerging possibilities in the development and function of regulatory T cells. Int Immunol 18: 991–1000. 16720616 10.1093/intimm/dxl044

[8] Woo EY, Chu CS, Goletz TJ, Schlienger K, Yeh H, et al (2001) Regulatory CD4(+)CD25(+) T cells in tumors from patients with early-stage non-small cell lung cancer and late-stage ovarian cancer. Cancer Res 61: 4766–4772. 11406550

[9] Liyanage UK, Moore TT, Joo HG, Tanaka Y, Herrmann V, et al (2002) Prevalence of regulatory T cells is increased in peripheral blood and tumor microenvironment of patients with pancreas or breast adenocarcinoma. J Immunol 169: 2756–2761. 12193750 10.4049/jimmunol.169.5.2756

[10] Wei WZ, Jacob JB, Zielinski JF, Flynn JC, Shim KD, et al (2005) Concurrent induction of antitumor immunity and autoimmune thyroiditis in CD4+ CD25+ regulatory T cell-depleted mice. Cancer Res 65: 8471–8478. 16166327 10.1158/0008-5472.CAN-05-0934

[11] Li J, Hu P, Khawli LA, Epstein AL (2003) Complete regression of experimental solid tumors by combination LEC/chTNT-3 immunotherapy and CD25(+) T-cell depletion. Cancer Res 63: 8384–8392. 14679000

[12] Dannull J, Su Z, Rizzieri D, Yang BK, Coleman D, et al (2005) Enhancement of vaccine-mediated antitumor immunity in cancer patients after depletion of regulatory T cells. J Clin Invest 115: 3623–3633. 16308572 10.1172/JCI25947 PMC1288834

[13] Ni Choileain N, MacConmara M, Zang Y, Murphy TJ, Mannick JA, et al (2006) Enhanced regulatory T cell activity is an element of the host response to injury. J Immunol 176: 225–236. 16365414 10.4049/jimmunol.176.1.225

[14] Lee H, Nho D, Chung HS, Shin MK, Kim SH, et al (2010) CD4+CD25+ regulatory T cells attenuate cisplatin-induced nephrotoxicity in mice. Kidney Int 78: 1100–1109. 20463654 10.1038/ki.2010.139

[15] Liu R, Xiong S, Zhang L, Chu Y (2010) Enhancement of antitumor immunity by low-dose total body irradiationis associated with selectively decreasing the proportion and number of T regulatory cells. Cell Mol Immunol 7: 157–162. 20140010 10.1038/cmi.2009.117 PMC4001896

[16] Kachikwu EL, Iwamoto KS, Liao YP, Demarco JJ, Agazaryan N, et al (2010) Radiation enhances regulatory T cell representation. Int J Radiat Oncol Biol Phys 81: 1128–1135. 21093169 10.1016/j.ijrobp.2010.09.034 PMC3117954

[17] Billiard F, Buard V, Benderitter M, Linard C (2011) Abdominal gamma-radiation induces an accumulation of function-impaired regulatory T cells in the small intestine. Int J Radiat Oncol Biol Phys 80: 869–876. 21345609 10.1016/j.ijrobp.2010.12.041

[18] Reddy J, Illes Z, Zhang X, Encinas J, Pyrdol J, et al (2004) Myelin proteolipid protein-specific CD4+CD25+ regulatory cells mediate genetic resistance to experimental autoimmune encephalomyelitis. Proc Natl Acad Sci U S A 101: 15434–15439. 15492218 10.1073/pnas.0404444101 PMC524444

[19] Carrigan SO, Yang YJ, Issekutz T, Forward N, Hoskin D, et al (2009) Depletion of natural CD4+CD25+ T regulatory cells with anti-CD25 antibody does not change the course of Pseudomonas aeruginosa-induced acute lung infection in mice. Immunobiology 214: 211–222. 19215803 10.1016/j.imbio.2008.07.027

[20] Couper KN, Blount DG, de Souza JB, Suffia I, Belkaid Y, et al (2007) Incomplete depletion and rapid regeneration of Foxp3+ regulatory T cells following anti-CD25 treatment in malaria-infected mice. J Immunol 178: 4136–4146. 17371969 10.4049/jimmunol.178.7.4136 PMC2235934

[21] Pidikiti R, Stojadinovic S, Speiser M, Song KH, Hager F, et al (2011) Dosimetric characterization of an image-guided stereotactic small animal irradiator. Phys Med Biol 56: 2585–2599. 21444969 10.1088/0031-9155/56/8/016

[22] Song KH, Pidikiti R, Stojadinovic S, Speiser M, Seliounine S, et al (2010) An x-ray image guidance system for small animal stereotactic irradiation. Phys Med Biol 55: 7345–7362. 21081818 10.1088/0031-9155/55/23/011

[23] Jankovic DL, Rebollo A, Kumar A, Gibert M, Theze J (1990) IL-2-dependent proliferation of murine T cells requires expression of both the p55 and p70 subunits of the IL-2 receptor. J Immunol 145: 4136–4144. 2258612

[24] Komatsu N, Hori S (2007) Full restoration of peripheral Foxp3+ regulatory T cell pool by radioresistant host cells in scurfy bone marrow chimeras. Proc Natl Acad Sci U S A 104: 8959–8964. 17494743 10.1073/pnas.0702004104 PMC1885610

[25] Timmerman R, Paulus R, Galvin J, Michalski J, Straube W, et al (2010) Stereotactic body radiation therapy for inoperable early stage lung cancer. JAMA 303: 1070–1076. 20233825 10.1001/jama.2010.261 PMC2907644

[26] Rusthoven KE, Kavanagh BD, Cardenes H, Stieber VW, Burri SH, et al (2009) Multi-institutional phase I/II trial of stereotactic body radiation therapy for liver metastases. J Clin Oncol 27: 1572–1578. 19255321 10.1200/JCO.2008.19.6329

[27] Sharp HJ, Wansley EK, Garnett CT, Chakraborty M, Camphausen K, et al (2007) Synergistic antitumor activity of immune strategies combined with radiation. Front Biosci 12: 4900–4910. 17569618 10.2741/2436

[28] Kajioka EH, Andres ML, Li J, Mao XW, Moyers MF, et al (2000) Acute effects of whole-body proton irradiation on the immune system of the mouse. Radiat Res 153: 587–594. 10790280 10.1667/0033-7587(2000)153[0587:aeowbp]2.0.co;2

[29] Chambers KA, Harrington NP, Ross WM, Filion LG (1998) Relative alterations in blood mononuclear cell populations reflect radiation injury in mice. Cytometry 31: 45–52. 9450524

[30] Gridley DS, Pecaut MJ, Nelson GA (2002) Total-body irradiation with high-LET particles: acute and chronic effects on the immune system. Am J Physiol Regul Integr Comp Physiol 282: R677–688. 11832386 10.1152/ajpregu.00435.2001

