# Supplementary material for: Partial Depletion of Regulatory T Cells Does Not Influence the Inflammation Caused by High Dose Hemi-Body Irradiation
Source: PLoS One. 2013 Feb 11;8(2):e56607. doi: 10.1371/journal.pone.0056607 (PMC3569437; doi:10.1371/journal.pone.0056607)
Supplement: Table S1 — Percentage of body weight change after irradiation and/or Treg cell depletion. (DOC) [file pone.0056607.s001.doc]

Supplementary Table S1.

Percentage of body weight change after irradiation and/or Treg cell depletion

| Treatment | % body weight change | | | |
| --- | --- | --- | --- | --- |
|  | Day 1 | Day 3 | Day 7 | Day 14 |
| Rat IgG+sham irradiation | 0.69 ± 1.87 | 3.42 ± 3.96 | 6.76 ± 3.22 | 9.61 ± 1.78 |
| PC61+sham irradiation | -0.75 ± 2.21 | 3.37 ± 3.21 | 4.23 ± 3.19 | 6.07 ± 3.44 |
| Rat IgG+irradiation | -5.23 ± 2.03 | -7.29 ± 2.45 | 1.74 ± 2.60 | -1.70 ± 1.74 |
| PC61+irradiation | -4.33 ± 1.51 | -5.54 ± 1.63 | -0.06 ± 1.95 | -2.06 ± 2.67 |

Note: Data are expressed as Mean ± SD (n = 4-16). Positive data indicates percentage of body weight gain after radiation; negative data indicates percentage of body weight loss after radiation. Two way ANOVA showed significant interactions over time between treatments (p<0.01). No differences were detected between PC61 + irradiation compared to Rat IgG + irradiation treatment group profiles over time (p=0.814). No significant differences were found between the PC61 + sham irradiation compared to Rat IgG + sham irradiation treatment group profiles over time (all p>0.05). All other pair-wise comparisons (Rat IgG + sham irradiation compare to Rat IgG + irradiation, Rat IgG + sham irradiation compare to PC61 + irradiation, PC61 + sham irradiation compared to Rat IgG + irradiation and PC61 + sham irradiation compared to PC61 + irradiation treatment groups) of group profiles over time were significant (p<0.05). All post hoc pairwise comparisons were performed with Tukey’s multiple comparisons. This is one representative experiment of three performed.
